# Abstracts and Gleanings

**Published:** 1880-11-20

**Authors:** 


### ABSTRACTS AND GLEANINGS.

**Ophthalmia Neonatorum.**—Dr. J. R. Wolfe, in a lecture on this subject (*Med. Times and Gaz.*, vol. ii., p. 259), says he has found that the larger number of the incurable blind owe their misfortune to the purulent ophthalmia of infancy. He urges upon practitioners the importance of abandoning the old routine treatment for this difficulty, and suggests the following measures. The diagnosis of the affection is as follows. On the third or fourth day after birth the baby's eyelashes are found stuck together with crusts forming at the borders, which are red. Next day the lids are more swollen, and the conjunctival sac filled with transparent, yellowish-colored serum and mucus. Within a week all the symptoms become intensified, and there is a copious discharge of pus, which runs over the cheeks. The eyelids are swollen so that they can only with difficulty be opened, and the cornea is found hidden and retracted in the purulent discharge. The cause of the trouble is that the child, in its passage from the uterus, has had its eyes inoculated with gonorrhœal or, possibly, leucorrhœal discharge from its mother's genital organs. The suppuration goes on in the eye until the reproduction of epithelium cannot keep pace any longer with the pus formation; then the covering becomes imperfect; the conjunctiva and subconjunctival tissues are attacked at the limbus; ulceration or abscess of the cornea ensues, ending in perforation; the eyeball bursts; the lens is evacuated; and the ball shrinks. Should the eye escape disorganization in some of the milder attacks, opacity of the cornea is left behind, causing strabismus, amblyopia, nystagmus, or opacity of the lens-capsule (capsular cataract).

If the old-fashioned, deleterious treatment is followed, which consists in dropping a solution of argenti nitrat. (gr. x ad ℥j) into the eye, the effect is either that the pus washes away the solution, rendering it innocuous (for it never touches the diseased surface), or it irritates the cornea, denuding it of its protective epithelium; the cornea ulcerates, or an abscess is formed, leading to the disorganization just referred to. Meanwhile, the eyelids swell so that the ball cannot be examined, and when the swelling goes down the eye is found to be gone.

Dr. Wolfe's procedure is as follows:

1. When seen in the first stage, before the purulent discharge has set in, the patient's head is placed on a towel and secured on the doctor's knees. The lids are then everted, singly or together, and, after cleaning them with dry lint, he touches the conjunctival surface with lint dipped in this solution:

℞. Boracis.........................................gr. x.

Aq. rosæ.........................................f ℥ j.

Aquæ ad.........................................f℥ vj.—M.

One dessertspoonful in two ounces of warm water.

He then puts a few drops of the solution of atropin upon the conjunctival surface:

℞. Atropiæ sulph.......................................gr. j.

Aquæ,...............................................f℥ ij.

Glycerinæ,..........................................fʒ ss.—M.

The application is repeated three times a day. The atropin has an antiphlogistic effect upon the inflamed surface. and also, by dilating the pupil, relieves the tension of the eyeball. Dr. Wolfe never uses cold applications, nor does he employ ointments to keep the lashes from sticking together; washing with warm water is better. Dry lint is then applied to the lids and secured by an immovable bandage. The case is watched carefully.

2. When the case is found to be unmistakably one of purulent ophthalmia, the lids are covered one after another, dried as before; a few drops of the solution of atropin dropped in, the surfaces touched with a stick of argenti nit. two parts, potass. nit. one part, and a few more drops of atropin put upon the cauterized surface. When the conjunctival surface is bleeding, (a favorable symptom), it is dried with lint and the cauterization repeated. The whole conjunctiva is touched, and also the *cul-de-sac.* He bathes it with lint and warm water, and covers the eyes with dry lint and a bandage. If one eye only is affected, the other is closed with court-plaster and covered with lint.

3. When called to see a case in the stage of advanced suppuration, say of three or four weeks' standing, the eyelids must be opened with great care, as the eyeball may be ruptured. If the cornea is found intact, the atropin and nitrate of silver pencil are to be used.

4. When an ulcer of the cornea or an abscess has already formed, it is the more urgent to use the nitrate as the only weapon to combat the disease. When the cornea is not actually ruptured, Dr. Wolfe generally manages to arrest the progress of the disease, and save it even if it is found in the process of softening or with an abscess. Such cases should be seen daily. In public hospitals or dispensaries Sundays must not be excepted, for one day's neglect may prove disastrous.—*Med. Times.*

**Gangrene from the Use of Ergot.**—J. Z. Van de Warker, M.D., of Davenport, Neb., writes:

Armanus Nott, age 20; temperament, nervo-bilious; school teacher. While attending school at Winimac, Ind., was taken sick with typho-malarial fever. Was treated five days by Dr. Thompson; was then brought home to his father's, near Medaryville, Ind., when I was called to take charge of the case. Found patient raving with typhomania; pulse, 136; temperature, 101°; tongue covered with brown crust in center and at the base, tip and edges bright red; teeth covered with sordes; petechia and sudamina well developed. I made the usual prescription to meet the indications, with the following to relieve the cerebral excitement:

℞ Hydrobromic acid.......................................ʒ i.

Sat. tinct ergot.........................................℥ ss.

Syrup lemon..............................................℥ ii.

Syrup acacia, qs. ad.....................................℥ iv.

M. Sig. Give one teaspoonful every three hours until delirium abates, then to be continued every six hours, to keep the patient quiet and procure sleep. The medicine acted like magic in controlling the mania; it was continued eight days, (to the fifteenth of the disease), when a new phenomenon appeared—a small ecchymosed spot on each foot, on the instep, about the size of a nickel five-cent piece. The spots appeared to have been bruised by the points of the thumbs of the hands of the nurse, who had been bathing his feet. At that time I gave it no further thought. In the afternoon of the same day was called in a hurry; found the patient *sinking* and very weak. The spots on the feet had spread to the knees, having the look and smell of *gangrene of the dermic tissue.* I gave stimulants and antiseptics internally; applied smart-weed, *steaming hot*, to the extremities to arouse the circulation, the application to be renewed every hour till the parts should become warm; then the smart-weed to be taken off and a poultice applied, composed of linseed meal, prepared with a decoction of fresh roots of the baptisia tinctora as strong as could be made, to be removed as often as every two hours, while there was any of the cadaverous smell or dark color remaining. I called next morning; patient was much improved in every way; the extremities were warm; the color nearly all removed, except a slight orange tinge on the limbs, and a slough of the derma on the instep of each foot about two inches in diameter. I concluded that the gangrenous condition was the sequel of the long-continued use of the ergot, so I promptly discontinued it, and prescribed antiseptic tonics. The patient rapidly improved and made a good recovery. My object in presenting the above case is two-fold: First, the use of hydrobromic acid, with ergot, in controlling cerebral excitement *and mania*, which it appears to control with certainty and promptness equaled by no remedy now known. Second, the danger of its long-continued use in producing capillary congestion and gangrene, as in the above case.

I have given as briefly as possible the main features of the case, and, in conclusion, will only say that, in controling all cerebral excitement and determination of blood to the brain, *even in concussion*, I have found no remedy equal to the above prescription in any and all cases where the "*regular*" *would use his lancet.—Chicago Med. Times.*

**The Prevention of the Spread of Typhoid Fever.**—The following case I have taken from an admirable report of it which appeared in the Popular Science Monthly for February, 1879. I have taken it from just without the limits of our State, because the accuracy of the report brings out a point in the ætiology which I have been unable to discover in any recorded instance of a similar occurrence among us.

In the city of S., in the State of New York, in a clustering group of thirteen houses on the outskirts of the town, a case of typhoid fever broke out on the 8th of September, 1876. The next occurred in the second house beyond, on the 4th of October, *twenty-six days later.* The disease then spread from house to house, until seven of the thirteen had been invaded, with a total result of seventeen cases and three deaths. The reporter distinctly states that on the 20th of September, after a hot and dry time, a tremendous storm of rain occurred, which filled and overflowed the privy vault into which the excrementitious matter of the first case was thrown, scattering the material which it contained all over the surface of the ground and into the neighborhood of the well from which all the families that suffered took their drinking-water; and, further, that none of the families in the group who did not use this well suffered.

These are typical examples of the spreading of typhoid fever as it has occurred among us. They illustrate the fact, to which I have alluded, that it spreads from a center first established by a single case. They illustrate also the fact that the establishment of such a centre requires time. They suggest by application that it is by means of the intestinal discharge that the infective centre is established.

I turn now to the presentation of a theory that such occurrences may be prevented by disinfection of these discharges, and to a brief consideration of some of the testimony supporting it. This testimony comes to us from various sources, and from authorities who differ somewhat among themselves as to the nature of the disease, and still more to the nature of the morbific agent by which it is propagated, but not at all upon the importance and value of disinfectant methods.

So far as I know, it is to the late Dr. William Budd, of England, that the medical profession and the public are mainly indebted for the theory of the prevention of the spread of typhoid fever by the disinfection of the intestinal discharges, and for the first practical suggestion of methods by which it may be carried out. The idea was, I think, with him. It was certainly through his earnest advocacy that it was first brought prominently into public notice, almost twenty-five years ago. His occasional contributions to the medical press upon this and kindred topics have made his name familiar, but he is probably best known among us by his elaborate work upon Typhoid Fever: Its Nature, Mode of Spreading and Prevention, which was given to the public in 1873.

Dr. Budd's attention was first called to the subject in 1839, by a terrible outbreak of typhoid fever in the little village of North Tawton, Devonshire, where he was then residing, and where, as a young practitioner, he was just beginning his professional life. North Tawton was a country town of only eleven or twelve hundred inhabitants, and its people were mostly engaged in agricultural pursuits. Dr. Budd was born there, and had grown up among the people. He knew them all personally. Moreover, he was, medically, the sole possessor of the field. All the cases of the disease passed under his immediate observation and care. The fever broke out in this secluded place in the second week in July, 1839, and before November eighty of the inhabitants had suffered by it. It furnished a typical illustration of the spreading tendency of the disease. Whole families were, one member after another, prostrated. It passed from house to house, and pervaded the place. Persons taken sick there left for their homes in neighboring towns, and carried the disease with them to new localities.

Opportunity more favorable for the study of such occurrenees could not have been presented, and Dr. Budd devoted himself to the task with enthusiasm. He traced the course and relation of events, observed everything in the living and the dead, and kept accurate notes of all. The whole experience evidently made a profound impression upon his mind, and gave a strong direction to his subsequent studies. He passed, with lapse of years, from the little country town to larger spheres of practice and usefulness and to great eminence in the profession, but always maintained to the end of life a continued and increasing interest in the great subject which had so signally attracted his early attention. To use his own expression, he seems to have been from the beginning "possessed with a burning desire to devote the best powers of his mind to a discovery of the means by which such calamities may be prevented."—DR. GAGE, *in Boston Med. Jour.*

**Treatment of Hemorrhoids by "Crushing."**—Mr. Pollock, of St. George's Hospital, London, employs an operation for hemorrhoids, which he terms crushing, and for which he claims the advantage over the ligature and the clamp and actual cautery, of being much less painful while it is equally effectual. The painfulness of the two last-named operations has always been a great objection to their employment, and while considering whether any modified process likely to be followed by less pain could be substituted for them, Mr. Pollock's attention was drawn to the known fact that any thorough and instantaneous destruction of a part is usually comparatively painless. It occurred to him, then, that if a pile could be rapidly and effectually destroyed at the base, by some instrument which would crush the part included in its bite, the vessels of the crushed portion would not be very liable to bleed when the surface of the pile would be removed, and, the nerves being bruised by the proceeding, the pain would probably be trifling. Some two or three years ago he began to put these views in practice, and the experience with the operation has been amply satisfactory. In the earlier operations he was often not as successful in preventing hemorrhage as was desirable, probably on account of defective construction of the clamp, or of taking up in its grasp too much of the tissues of the pile at once. Still, the hemorrhage was never alarming and was always easily controlled by two or three ligatures. In his late operations with an improved clamp, occasionally one or two small vessels have bled after the base of the pile was removed from the grasp of the instrument. The hemorrhage in these cases would probably have ceased spontaneously, but a ligature was always applied to any suspicious point for the sake of cleanliness, and to avoid giving any cause for alarm. From the very first, however, the operation proved very successful in one important particular, viz., the pain following it was very insignificant. The slough shed after the application of the crusher is very thin, and the œdema is slight compared to what often occurs after the other operations. The final results in the cases operated on have been fully as good as those obtained by the ligature and the actual cautery.

Mr. Pollock uses a powerful clamp made for the purpose by Wright & Co., of London. The steps of the operation are as follows: The patient is prepared for the operation in the usual way; when he has been brought under the influence of ether, he is turned on his left side and his right leg is well flexed and fixed with a strap, which is carried under the knee and around the neck. The pile to be removed is then well drawn down, and the clamp is applied to its base and at once tightly and firmly closed by the action of the screw at the end of the handles. The portion of the pile which protrudes *inside* the lips of the clamp is then to be removed with curved scissors. The clamp should be kept applied to the stump of the pile for about a minute longer, or for a still longer period if the pile be large and thick. The process is of course to be repeated according to the number of masses to be got rid of.—*The Lancet*, July 3, 1880.

**Transplantation of Skin From the Sheep to the Human Body.**—A Chicago correspondent writes of a curious experiment at the Cook County Hospital in that city, by Dr. E. W. Lee, by which it is sought to transplant skin from the body of a sheep to that of the human subject. The case has excited much interest in the professional circles which have been cognizant of the operation.

The subject of this experiment (that is the human subject) is a girl about ten years of age, who sustained an extensive burn on the back a year and a half ago. A large granulating ulcer remains, despite all efforts to induce healing. Skin grafting has been faithfully practiced, but without success. The child has of course been obliged to lie prone most of the time, and has become greatly reduced. A few weeks ago an attempt was made to transplant a flap from the thigh of her older brother, but the flap sloughed. That failing, Dr. Lee began at once to experiment with flaps and dissections from the sides of sheep. His first subject, a lamb, nearly full grown, was lost, soon after the dissection of the flap, from the shock of the operation. He next operated on two other lambs, dissecting up two moderate-sized flaps from each, placing oiled silk beneath them to prevent adhesion, and dressing them antiseptically. These animals were then turned out to grass in the hospital yard, and were also fed on milk, with occasionally a small admixture of whiskey. They took to this diet with avidity. After several days—nearly two weeks—had elapsed and the animals were vigorous, the operation of the application of the flaps of one of them was made. A new flap was disected from between the two already made, and applied in the same manner as the others. The new flap has made a firmer adhesion than the old ones. The lamb is fastened in the standing posture, in a wood cage, its body being securely fixed and sustained by plaster-of-Paris bandaging of its limbs and quarters. Perfect coaptation and perfect immobility are secured. The patient has improved in appearance and general condition since the operation and the lamb shows no signs of failing health.

At the date of writing six days had elapsed since the operation and unions of all the flaps seemed to be perfect. Of course when they come to be separated from the lamb they may suffer and perhaps slough, but the experiment has demonstrated the fact that the skin of the sheep will adhere to a granulating surface on the human body.—*Mich. Med. News.*

**Treatment of Diphtheria.**—In a recent number of your medical journal a physician in the State of Indiana requested that some of his medical brethren of experience would give for the treatment of diphtheria a formula that had been attended with the greatest success. Having been engaged in the practice of my profession nearly a score of years, and having varied and extensive opportunities of investigating and treating diphtheria in several localities, and having never yet lost a single patient, I therefore do not hesitate to lay my method of treatment before the profession. Were I to view the pathology of diphtheria in the light of my friend, Prof. Bristowe, I, like he, should hesitate in adopting any permanent routine of treatment, but, on the contrary, I am satisfied that diphtheria is a disease of constitutional origin, and that the local symptoms are but an effect from a constitutional cause, that the disease assumes an æsthenic type from the commencement, and to successfully fight the disease we must attack it at its centre of operation, which is in the blood. I must not, however, in this short article, engage the reader's attention either on the pathology or morbid anatomy of the disease before us, but would request the reader to investigate the doctrines promulgated by J. S. Bristowe, of London, England, and Prof. Flint, of our own country, excepting the similarity of pseudo membranous croup and diphtheria as quoted by Prof. Bristowe. When called to see a case of diphtheria, it is just as important for the practitioner to investigate the condition of all the secretions and excretions as under any other circumstances. I nearly always prescribe a mercurial cathartic at first, and in the course of several hours administer one tablespoonful of Rochelle salts in one-half tumbler of water at two doses; I then administer muriated tincture of iron with tonic doses of quinine sulphat. in sweetened water every two or three hours, regardless of increase of temperature. If the patient has fever, I control it with the tincture of veratrum viridi, given in medicinal doses according to age; I also give Rochelle salts in small doses, largely diluted with water, two or three times a day; also put the patient on the following syrup mixture:

℞ Simple syrup.......................................... ℥ iv.

Ammonia murias.......................................... ʒ i.

Pulv. ipecucuanhae.......................................... grs. x.

Pulv. glycyrrhiziae.......................................... ʒ ii.

Directions: Pour the simple syrup, while hot, on the other ingredients; cover over, and when cold give teaspoontul every two or three hours.

Now, for local treatment without discussing different methods of medication:

℞ Pure water.......................................... ℥ iv.

Bromo chloralum.......................................... ʒ ss.

Saccharum album.......................................... ʒ ss.

Directions: Gargle as in the preceding.

I denounce the practice of swabbing and the use of cauterants. If the dyspnœa is great, I sometimes give an emetic of ipecac in warm water, and always bathe the throat externally several times with spirits turpentine with as much camphor dissolved as it will take up.

Mr. Editor, I have aimed to give the treatment in as practicable and plain a way as I could, so that the possibility of mistake may be avoided. You will perceive that all of the medicines I use support, except the veratrum viride.—*Cin. Lancet.*

**The Iodine Treatment of Intermittent Fever.**—Dr. Gibbons, in San Francisco Medical Society, says: The proper place of iodine in the treatment of intermittent fever is not as a prompt anti-periodic, to prevent the immediate recurrence of the chill, but as an alternative, to be administered after the interruption of the paroxysms, for the purpose of preventing their return.

Twelve drops of the tincture three times a day, an hour after meals, was the formula. In a certain proportion of cases, say one-third at least, there was no recurrence of the paroxysm after instituting the treatment. Several old cases strongly marked with the malarial cachexy, and which had been repeatedly and freely dosed with quinine, to my surprise recovered with no other treatment. Indeed, the remedy is often most efficacious in such cases. But in the majority of patients the paroxysm returned, in spite of the iodine.

Having satisfied myself that the agent could not be depended on alone, I then adopted a mixed treatment, first breaking the chain of continuity in the paroxysms by a cinchona anti-periodic, and then instituting the iodine treatment exclusively. This course was successful, almost without a solitary exception. I may safely say that the chill did not return during the use of iodine in more than one case in fifty. In some instances of threatened return the dose was increased up to fifteen drops.

The toxic effects of the iodine, when they appeared, were developed mostly in the stomach and digestive organs. To loss of appetite and gastric distress were added a variety of unpleasant sensations, of which the patient complained without being able to define them accurately. One instance assumed quite a serious form, and continued for a number of weeks. In view of these consequences, I adopted the plan of suspending the iodine for a short time, after about two weeks' use; and when the cure was well-established continuing it for a while on alternate weeks only.

As to the *modus operandi* of iodine in this disease, I have only to say that it appears to exert a specific action on the malarial condition. Probably it does this through the liver and spleen, as congestion and enlargement of those organs will often disappear under its use. Without doubt, we are to take in account its well known power of promoting absorption and stimulating the glandular organs.—*Pacific Medical Journal.*

**Concentrated Extracts—Therapeutics.—Uses of Boracic Acid.**—"Considering the well-known antiseptic properties of boracic acid, it is exceedingly curious how little it has been administered as an internal remedy. Its effect in diphtheria, both locally and internally, is very marked, and the following statement by Drs. Cossar Ewart and Malcolm Simpson proves in a pretty conclusive manner the action it has upon the disease germs: 'Pieces of membrane which had been brushed with a saturated solution of boracic acid, when placed on the warm stage of the microscope showed the characteristic bacilli; but these were absolutely innocuous, and instead of lengthening into spore-bearing filaments, micrococci bacterium termo or torula appeared in their stead. By the use of the acid, the disease was shortened, and the other members of the family were protected from infection.' In the treatment of *puerperal fever*, combined with sulphuric ether (which is also an antiseptic), and, when it has been found necessary, a little tincture of opium, it has given more decidedly beneficial results than anything with which I am acquainted. I feel certain that it ought to hold an important place in the treatment of carbuncular disease—erysipelas, cholera, scarlat'na, enteric, typhus and intermittent fever—and in fact all those cases which are known to have a septic origin. From what I know of its power in combating the action of disease-germs, I cannot help thinking it would materially lessen, not only the intensity, but also the duration of the various eruptive fevers. I incline to this belief very strongly; time will quickly show whether it is correct or not. It is but sparingiy soluble in cold water; an ounce will only take up about eighteen grains, but a drachm of boiling water will dissolve about five grains. The dose is from five to fifteen grains. It has one particular recommendation, and that is its tastelessness."—*St. Louis Clinical Record.*

**Syphilis in Relation to Marriage.**—Dr. Alfred Fournier has recently published a book, called "Syphilis et Mariage," which contains the substance of what he has been teaching in regard to this subject for some years. It may be summed up as follows:

The physician who is questioned by a man who has had syphilis as to the propriety of his marrying, has a very grave responsibility imposed upon him. If he decide amiss, he may, on the one hand, let a man marry only to infect his wife and beget a diseased offspring; or, on the other hand, forbid a marriage which would be perfectly or reasonably safe and close the door against all the advantages which would follow it. When he (Fournier) thinks of the happy couples and the healthy children who owe their happiness and existence to his permit, he cannot but think it is a grave error to say a syphilitic should never marry

If, then, this be sometimes permissible, when is it so? The answer he gives is:

1st. In the absence of actual syphilitic manifestations; 2d. After a considerable time from that at which the disease was acquired; 3d. After a period of immunity since the last outbreak; 4th. When the disease has been of a non-menacing character; 5th, After adequate specific treatment.

The first condition is absolute and inflexible; the second admits the exercise of a certain discretion, though it should not be less than three or four years, and is effected by the fifth; the third condition, the period of immunity, he thinks should be at least from eighteen months to two years. The character of the disease may be shown to be non-menacing by the patient's general condition or by the lightness of his eruptions; while an early affection of the nervous system or the viscera is a very grave sign. By adequate specific treatment, Fournier means active and curative, not timid and indifferent, doses of mercury and iodide of potassium. These are to be used methodically and as indicated in the different stages and degrees of the disease.

This much in regard to the conditions precedent to permitting the marriage of a man who has had syphilis. Fournier, in common with most men who have made a special study of syphilis, considers this a curable disease. The old teaching, that it is ineradicable, and descends from father to son to the remotest generation, is abandoned by those who have the best right to positive opinions about it. It may be remarked, in passing, that Fournier is of those who believe in paternal heredity: that a father may beget a syphilitic child without infecting the mother. But in most cases it is first to the wife that the danger comes, and secondarily to the offspring.

Nevertheless, it is a great thing to believe that both may escape; that, in a fairly healthy person, syphilis is but a grave disease, not an incurable one; that care and patience and appropriate treatment may restore the subject of it to the ranks of healthy men, fit him for marital relations and give him the opportunity to beget sound and sane children that shall not bear the stamp of their father's disease. Such views are no longer novel, and are familiar enough to special students, though they have not yet established themselves in the minds of all who have the shaping of medical opinions. There are still men of eminence and learning who repeat what used to be taught in regard to this matter. Their belief is entitled to the greatest respect, and a presumptuous rejection of it might lead to very sad results. Still the actual experience of men who have made syphilis the study of their lives must carry the greatest weight, and may be rationally accepted by those who wish, with the best light they can obtain, to decide the important questions that may be put to them by men whose happiness will depend upon the answers they then give.—*Specialist and Intelligencer.*

**The Non-Specific Treatment of Gonorrhœa.**—The efficacy of the abortive and specific treatments of gonorrhœa cannot be questioned for a moment; but, unfortunately, they are often disgusting and distasteful to the patient. The disagreeable odor perceptible about a person taking copaiba, cubebs or sandal wood, is frequently apparent, not only to the patient himself, but also to those with whom he comes in contact. This is unpleasant to a patient, no matter to what class he may belong. Further, there have been instances in which the fear that the medicinal odor might disclose his ailment has produced in the patient such an amount of anxiety that the mental condition was as distressing to him as the disease itself. To avoid these difficulties, the following is the plan of treatment I have used in a number of cases, with good results.

In the acute stage of the disease, that is, before the discharge is well marked, and when there exists an intense, agonizing, burning pain upon micturition, I have been in the habit of making the constitutional treatment predominate over the local; that is to say, restricting the diet, regulating the bowels, ordering rest, if necessary, and giving internally:

℞ Potass. acetat.........................................ʒ ij.
Uvae ursi.........................................ʒj. M.

Sig.: To be put into one pint of boiling water and a wineglassful (f. ℥ ij.) to be taken every two hours, in flaxseed tea.

I order the inflamed organ to be kept well cleansed with hot or cold water, whichever is most agreeable to the patient, containing a small amount of salt or alum. Having continued this treatment for two or three days, I order, in addition, an injection:

℞ Zinci acetat., (vel sulphat.).........................grs. vj.

Glycerine.........................................f. ℥ ss.

Aquae rosae.........................................f. ℥ ijss. M.

Sig. : Use three or four times daily.

Before using, the patient should urinate, if possible, so as to cleanse out the uretha. If chordee or orchitis occur, it is treated with opiates and antiphlogistic measures.

The length of time to continue this course of treatment varies with the severity or mildness of the attack; but in no instance have I been led to feel that the disease had been prolonged, or that I had been guilty of an injustice to my patients in discarding the time-honored specific remedies.

There is one point upon which I lay great stress, viz: delaying the use of an injection for two or three days, or until the acute inflammatory condition has subsided and the discharge appears. The use of an injection in the first stage of gonorrhœa is very painful, and tends to increase, instead of allay, the existing inflammation; but if acetate of potash and uva ursi are first administered, the urine is rendered bland and less irritating, the inflammation subsides, the urethra becomes less sensttive, and the whole organ is in a far more favorable condition for the reception of local medication.

A utilitarian consideration in cases of this sort is, that if stricture should supervene where an injection has been ordered at the onset of the attack, the patient is likely to associate the stricture with the pain caused by the injection, and attribute it to unnecessary strength of the latter.

The internal treatment suggested, as compared with the specifics, meets all demands, and does not produce the unpleasant effects—nausea, cutaneous eruptions, renal pain, etc.,—attendant upon the administration of copaiba, cubebs and sandal wood.

The disgust expressed by some patients for the "specific treatment," and the decided objection of others to be placed upon it, have influenced me in adopting the method I have described, and I have never yet had cause to regret it.—DR. RONALDSON, *in Specialist.*

**Prussian Blue in Malarial Fever.**—Through the suggestion of a friend of mine, wrote Dr. W. I. Martin, I was led to the use of the above article in some stubborn cases of intermittent fever, more particularly that form termed "dumb ague or chronic chills." Now, after the use of it for several years, I have found it an efficient remedy, and have rarely been disappointed in being able to effect a cure. It must be given in pretty large doses, much larger than is directed in our books, to have this desirable effect, say ten grains three times a day. This amount, in my hands, has proved sufficient for the adult. In looking at the article chemically, we might fear using such large doses, but, after experience, I have seen nothing bad result from it whatever. Given in powder, dropped upon the tongue and washed down with a little water, is the most eligible way of administering it. The taste is somewhat similar to that of powdered charcoal, and but few complain of it being unpleasant to take; in this way even children take it readily. Given in pillular form, a dose would make three pretty large pills; this amount having to be taken three times a day, we would find but few that would submit to it. Prussian blue being a chalybeate, has the effect of that class of remedies, as well as that of an anti-periodic, and I found it to be most efficient in those cases where there is a

"run-down" or anæmic condition, and when a cure is effected it is more permanent than that from quinia. There are some cases where quinia will not make a permanent cure, even when given under the most approved plan, which, I believe, is after the chill has been arrested by it, to keep them from returning by giving a full dose the day preceding the seventh, fourteenth and twenty-first day following the last chill. Now, in these cases, where quinine has so failed, the ferro-cyanide of iron comes in as just the thing.—*Med. and Surg. Rep.*

**The Coca in Opium Habit.**—In your issue of May 29, I noticed an article from the pen of Dr. Palmer, upon coca as a possible antidote for the opium habit. At that time I had under my treatment Captain C., who was suffering from the morphine habit. He was wounded in the left leg at the battle of Nashville during Hood's raid through Tennessee, and had it amputated at the middle third of the thigh. He contracted the morphine habit to alleviate the intense pain, and continued it for several years. Five years ago he quit taking the drug, and abstained till last spring, when he went to Louisiana from Middle Tennessee, where his physician prescribed morphine, in connection with quinine, for the relief of malarial poisoning. The old habit soon returned with all its pristine force. When he came back, I found him in the condition described above—gloomy, despondent, and threatening to commit suicide.

As soon as I read Dr. Palmer's article, I determined to give the coca a fair test, and am able to report that the result was a most happy one. He has been using the coca *ad libitum* for more than a week, and now, instead of taking three grains of morphine several times a day, is entirely relieved of this habit, with all its distressing effects, and is happy, hopeful and cheerful.

I hope all other physicians will try this new remedy in cases of this kind, and report through the *News* and other medical journals, as I, for one, am deeply interested in the result.—J. G. CORE, *M.D., in Louisville Medical News.*

**Cause and Treatment of Bromidrosis of the Feet.**—Dr. Thin, in a paper on the cause of the bad odor sometimes associated with excessive sweating of the feet, (Brit. Med. Jour., vol. ii., 1880, p. 463), calls attention to the fact that this odor does not belong to the sweat itself, but is in the coverings of the feet. In a case experimented upon to verify this fact, which has been noted by Hebra, the hands of the patient, a young woman, which sweat profusely, were free from odor, while the feet gave out a disagreeable smell in moist weather, being quite inoffensive in dry, bracing weather. The soles of the shoes and stockings being subjected to the action of an anti-septic, the smell was entirely banished. It soon returned, however, and examination showed the stockings to be saturated with a secretion filled with bacteria. When, however, the stockings were immersed in a jar containing a saturated solution of boracic acid and dried, the smell disappeared. Taking this hint, these coverings were disinfected with the acid previously to wearing, with good result. To prevent the sodden, disagreeable smell of the shoe-soles due to this same cause, the patient was directed to get cork soles. Each pair of these, after having been worn a single day, were placed over-night in the boracic acid solution, and were the next day dried. On the third day they were again ready for use. The skin was also washed with the boracic acid solution, which hardened and refreshed it. The cure was very satisfactory.—*Med. Times.*

**Hysterics.**—Dr. William Goodell recommends the following for hysterics in young ladies:

"When you are called on to treat a young girl with a hysterical attack, there are three things which you had better do:

1. "Institute at once firm pressure in the neighborhood of both ovaries. This is very apt to quiet the patient at once.

2. "Administer an emetic. I have found that a woman who is well under the action of an emetic has not the opportunity to do anything else than be thoroughly nauseated. Give a full dose of ipecac with one grain of tartar emetic.

3. "And this method of controlling the spasm will often act charmingly: take a good sized lump of ice and press it right down upon the nape of the neck. This produces quiet by its powerful impression upon the nervous system.

"When the attack is entirely under control, the best method of preventing the occurrence of another attack is to administer a full dose of assafœtida—none of your small two or three grain doses, but ten grains all at once."—*Maryland Med. Jour.*

**Boracic Acid Injections in Gonorrhœa.**—For some months past I have observed the excellent results obtained by Prof. Seely in the dispensary of the Medical College of Ohio, in the treatment of cases of acute and chronic middle ear inflammations, and of purulent conjunctivitis, by means of boracic acid. These observations have led me to test its action in other parts of the body.

No better or wider field appeared to present than in cases of gonorrheal urethritis, so frequently occurring in the practice of every physician, and the treatment of which has, notwithstanding our large experience, remained very unsatisfactory.

The first case of this character in which I prescribed boracic acid, was that of a young man in the acute inflammatory stage of the disease, with abundant discharge, frequent and painful micturition and very troublesome chordee. Several of the more popular remedies had already been given without affording the slightest relief. After the first day's use of a one per cent. solution he was no more troubled by chordee. The pain attending micturition was much lessened after a single injection, and disappeared entirely upon a few repetitions. The discharge rapidly diminished in quantity and changed in character, but did not altogether cease for about a week.—*Country Practitioner.*

**Transverse Depressions on the Nails.**—Dr. James Sawyer, in a note to the Lancet, agrees with Dr. Duckworth that "there is a rather more rapid formation of nail than that of two complete growths in a year." From his own observations, he should say that from three to four months are usually occupied in the passage of a furrow from the lunula to the end of the nail. These grooves are very common. They are sometimes to be seen on all the finger-nails; often they occur only on the thumb-nails. If a person's nails be free from transverse furrows, we may conclude, almost with absolute certainty, that he has not had a serious illness in the last three or four months. He has found three or four of these depressions, equidistant and parallel, on the thumb-nails of women who are the subjects of dysmenorrhœa—a furrow making each painful "period."—*Druggists Circular.*

**Hodge Pessary.**—Dr. E. H. Trenholme, in London Obstetric Journal, from his own experience and study of the Hodge pessary, concludes:

1. Variously modified it is an efficient and most admirable instrument for sustaining a recto-dislocated uterus, and that to any desired elevation in the pelvis.
2. Even a large pessary, filling and distending the vagina and taking pressure on the floor of the pelvis, can be worn with comfort and ultimate curative results by the proper use of the postural treatment, together with the inflation of the vagina by elevating the floor of the pelvis, while in that position.
3. The curative forces operating upon the uterus are resultants of (*a*) the elevating power of the pessary; (*b*) the resisting force of the sacrum; (*c*) the weight of the uterus, now so high up as to gravitate forward and downward, and (*d*) the pressure of the abdominal viscera.
4. The vices of flexion and position being overcome, a permanent recovery may be looked for with certainty in from six months to a year from commencement of treatment.—*Obstetric Gazette.*

**Concerning Coca.**—There can be no question of the potency of coca, and in the hands of the intelligent physician much good may be expected from its use. But do take your trenchant pen and give us a slashing article against the indiscriminate use of it. You know a confirmed chewer of coca is called a eoquero. Among the Spanish Americans a coquero is considered hopelessly lost, with no prospect of reformation. Look at the picture drawn by Von Tschudi: "The inveterate coquero is known at first glance. His unsteady gait, his yellow skin, his dim and sunken eyes encircled by a purple ring, his quivering lips, and his general apathy, all bear evidence of the baneful effects of the coca juice when taken in excess."

Surely this picture is enough to startle any one; but I know that some cannot be startled if there is a prospect of satisfying an appetite; therefore, the greater need of a fiery warning in time, to the profession, as well as the masses, against the indiscriminate use of a drug which is so apt to be followed by the blasted and desolate life of the coquero.—D. H McDONALD, M.D., *in Louisville Med. News.*

**Treatment of Post-Nasal Catarrh.**—In the *London Med. Record,* which frequentla contains useful hints for the general practitioner, I saw lately a formuly for the treatment of post-nasal catarrh. It was, I believe, originally suggested by Dr. Duffin. It consists of oxide of bismuth, powdered gum acacia, and a small quantity of muriate of morphia. It should be well mixed, and then, if used as a snuff in severe coryza or post-nasal catarrh, it acts in a most charming manner. Cases of great severity and long duration have yielded to its influence after three or four days.—*Country Practitioner.*

